# Is Irritable Bowel Syndrome Considered as Comorbidity in Clinical Trials of Physical Therapy Interventions in Fibromyalgia? A Scoping Review

**DOI:** 10.3390/jcm10204776

**Published:** 2021-10-18

**Authors:** Paula Mª Rodríguez-Castillejo, César Fernández-de-las-Peñas, Francisco Alburquerque-Sendín, Daiana P. Rodrigues-de-Souza

**Affiliations:** 1 Department of Nursing, Pharmacology and Physical Therapy, Faculty of Medicine and Nursing, University of Córdoba, 14004 Córdoba, Spain; n72rocap@uco.es (P.M.R.-C.); falburquerque@uco.es (F.A.-S.); 2 Department of Physical Therapy, Occupational Therapy, Rehabilitation and Physical Medicine, Universidad Rey Juan Carlos, 28922 Alcorcón, Spain; cesar.fernandez@urjc.es; 3 Cátedra Institucional en Docencia, Clínica e Investigación en Fisioterapia: Terapia Manual, Punción Seca y Ejercicio Terapéutico, Universidad Rey Juan Carlos, 28922 Madrid, Spain; 4 Maimonides Biomedical Research Institute of Cordoba (IMIBIC), 14004 Córdoba, Spain

**Keywords:** fibromyalgia, irritable bowel syndrome, clinical trials, physiotherapy, eligibility criteria

## Abstract

Evidence supports the presence of comorbid conditions, e.g., irritable bowel syndrome (IBS), in individuals with fibromyalgia (FM). Physical therapy plays an essential role in the treatment of FM; however, it is not currently known whether the IBS comorbidity is considered in the selection criteria for clinical trials evaluating physiotherapy in FM. Thus, the aim of the review was to identify whether the presence of IBS was considered in the selection criteria for study subjects for those clinical trials that have been highly cited or published in the high-impact journals investigating the effects of physical therapy in FM. A literature search in the Web of Science database for clinical trials that were highly cited or published in high-impact journals, i.e., first second quartile (Q1) of any category of the Journal Citation Report (JCR), investigating the effects of physical therapy in FM was conducted. The methodological quality of the selected trials was assessed with the Physiotherapy Evidence Database (PEDro) scale. Authors, affiliations, number of citations, objectives, sex/gender, age, and eligibility criteria of each article were extracted and analyzed independently by two authors. From a total of the 412 identified articles, 20 and 61 clinical trials were included according to the citation criterion or JCR criterion, respectively. The PEDro score ranged from 2 to 8 (mean: 5.9, SD: 0.1). The comorbidity between FM and IBS was not considered within the eligibility criteria of the participants in any of the clinical trials. The improvement of the eligibility criteria is required in clinical trials on physical therapy that include FM patients to avoid selection bias.

## 1. Introduction

According to the American College of Rheumatology (ACR), fibromyalgia (FM) is a chronic condition associated with widespread pain, fatigue, sleep problems and tenderness [1]. With a worldwide prevalence of 2–3% [2], its prevalence in Spain is 2.4% [3]. Although the diagnostic criteria have evolved from the exclusive presence of generalized pain and pain on palpation at specific locations [4] to the inclusion of questionnaires on pain perception and distress [5], its prevalence has increased as diagnostic criteria evolved [6]. Nonetheless, this pain condition is under-, over-, or misdiagnosed [7]. Regarding the distribution by sex, FM is more frequent in women than in men (female: male ratio 9:1) [8]. Although FM is considered a noninflammatory generalized musculoskeletal pain condition associated with fatigue and sleep disturbances, many patients also exhibit cognitive dysfunction (e.g., brain fog), mood disorders, or intestinal comorbidities, e.g., irritable bowel syndrome (IBS) [9].

The etiology and pathophysiology of FM are not well understood [2]. Current theories describe a potential disorder of nociceptive pain processing and central sensitization as the main pathophysiological mechanism [10]. Current evidence shows that individuals with FM usually exhibit several medical comorbidities, e.g., diabetes mellitus, IBS, or mood disorders [11]. Clinicians routinely overlook somatic symptoms, however, if comorbidities are taken into account and treated appropriately, the severity of the symptoms in patients with FM would be reduced [12].

Specifically, IBS is present in 46.2% of people with FM [13], whereas FM occurs in 12.9% to 31.6% of patients with IBS [14]. Both conditions share a wide variety of symptoms such as fatigue, insomnia, anxiety or depression, as well as a predilection for the female gender. As in FM, the pathophysiology of IBS is not understood. There is a hypothesis suggesting a dysregulation of the brain−gut axis resulting in a state of generalized hyperalgesia (also known as central sensitivity disorders) leading to increased excitability of central nociceptive pathways and inhibition of descending pain modulation [15]. This hypothesis could explain the comorbidity presentation between FM and IBS and also the presence of common symptoms.

This similarity between both syndromes should create the need to more exhaustively define the selection of participants with FM in clinical trials [12,13]. Treatment of FM is clearly multifactorial, but it seems that physical therapy has shown to be beneficial for these patients. Several meta-analyses support that physical therapy interventions are effective for reducing symptoms and for improving health-related quality of life in individuals with FM, exercise probably being the most effective [16,17,18]. The main target of physical therapy interventions is the musculoskeletal system, the presence of a comorbid visceral disorder, e.g., IBS, could lead to the necessity of different therapeutic strategies.

Meta-analyses represent the highest level of evidence supporting or refuting a therapeutic intervention, but they are based on randomized clinical trials. The influence of relationships between FM with psychological and somatic-visceral conditions could lead to uncertainty regarding the etiology of symptoms and may influence those clinical outcomes. If medical comorbidities are ignored in the selection criteria of clinical trials, this may limit the potential benefits of physical therapy, which mainly targets musculoskeletal symptomatology. In fact, it has already been identified that the comorbid relationship of IBS with temporomandibular disorders [19,20] is underreported when designing clinical trials evaluating the effectiveness of physical therapy [21]. It is, therefore, reasonable to consider that the relationship of FM with IBS could also influence clinical outcomes. No study has previously investigated this topic in clinical trials including individuals with FM. The objective of this scoping review was to identify whether the presence of IBS has been considered in the participating selection criteria of “relevant” clinical trials evaluating the effects of physical therapy in individuals with FM.

## 2. Methods 

This scoping review was conducted following the methodological framework suggested by Arksey and O’Malley [22] consisting of: 1, identify the research question; 2, identify relevant studies; 3, study selection; 4, data extraction; 5, compiling, summarizing, and reporting results. Additionally, it also adheres to the adjusted items for systematic reviews and meta-analysis extension for scoping reviews (Prisma-ScR) and it has been registered in The Open Science Framework Registry (https://osf.io/ns35d (accessed on 20 December 2020)).

### 2.1. Research Question 

The research question of the current scoping review was: has the presence of visceral disorders, e.g., IBS, been considered within the selection criteria of “relevant” clinical trials investigating physical therapy interventions in individuals with FM?

### 2.2. Identifying Relevant Studies

The literature search was conducted in the Web of Science (WOS) database from the inception of the database to 22 December 2020. The search was conducted by two different assessors and was limited to high-quality clinical trials including humans. No language restriction was applied. A combination of the following terms employing Boolean operators was used for the search: “fibromyalgia” AND, “physical therapy” OR “physiotherapy” OR “exercise” OR “manual therapy”. 

### 2.3. Study Selection

In this review, the PCC (Population, Concept and Context) mnemonic rule was used to define the inclusion criteria.

Population: Men or women diagnosed with FM according to 1900 ACR diagnostic criteria [4], 2010 ACR diagnostic criteria [23] or modified 2010 ACR classification criteria [5]. Alternative diagnostic criteria for FM were also accepted if properly described in the paper.

Concept: Randomized clinical trials evaluating any type of physical therapy intervention, alone or in combination with others, in individuals with FM. 

Context: Articles that met one of the following criteria were considered “relevant”: 1, most cited clinical trials from the selection; or, 2, clinical trials published in high impact journals, e.g., of the first quartile (Q1) of any category of the Journal Citation Reports (JCR) evaluated in the year of publication of the study, according to WOS (JCR criterion−impact factor).

The selected studies were identified independently by two investigators considering the title and abstract. For potentially eligible articles, the full text of the article was read. If both researchers disagreed on the inclusion/exclusion of any of the articles, a third researcher decided whether to include/exclude it. All data were saved and managed through Microsoft Office.

### 2.4. Data Extraction

From selected studies, the following information was extracted following a standardized form: number of authors affiliated with a clinical institution (e.g., hospital, private clinic, or health center), number of authors affiliated with a nonclinical institution (e.g., university or research center), total number of citations in WOS, PEDro score, study objectives, sample size, characteristics of participants (sex distribution, mean age), and inclusion and exclusion criteria for participants selection.

### 2.5. Methodological Quality 

The Physiotherapy Evidence Database (PEDro) scale score was used to determine the methodological quality of clinical trials. This scale contains 11 items, which can be scored as absent (0) or present (1), except for the first item that refers to the external validity of the study. Each item is scored from 0 to 10 points. The PEDro scale is a valid, reliable and widely used tool for rating the methodological quality of clinical trials [24]. Studies were considered to be of high quality if they obtained at least 5 points on this scale. The score for each article was extracted from the PEDro database, except for those that were not evaluated by the database. In this case, they were evaluated by two researchers following the guidelines established by the PEDro scale [25].

## 3. Results

### 3.1. Study Selection

**Figure 1:**
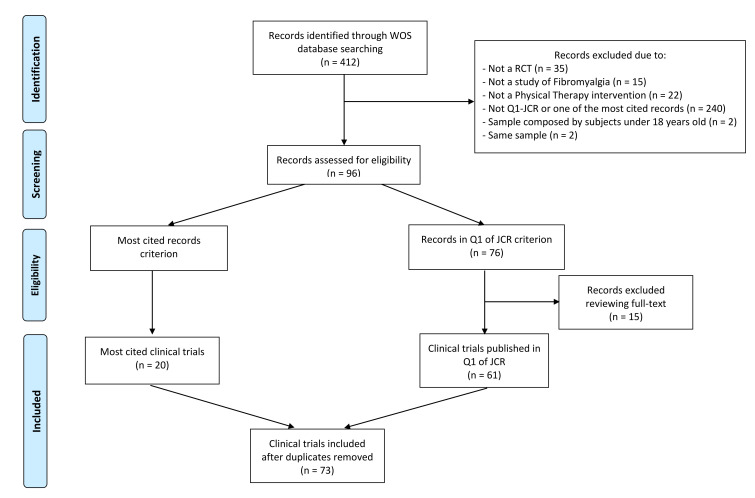
PRISMA extension for scoping reviews (PRISMA-ScR) flow diagram. WOS: Web of Science; RCT: randomized clinical trial; JCR: Journal Citation Reports.

The search identified a total of 412 articles. Of these, 316 were excluded because they did not meet any of the criteria. Of the remaining 96, the full text was accessed, after which 15 articles were excluded. Finally, 20 trials according to the citation criteria and another 61 published in Q1 JCR journals were included. Eight of the selected articles met both criteria; accordingly, a total of 73 different clinical trials were included (Figure 1).

### 3.2. Study Characteristics

Data extracted from the most cited trials are shown in Table 1, data from trials published in Q1 of any category of the JCR are summarized in Table 2, and those fulfilling both criteria are described in Table 3. The total number of citations from those trials included in Table 1, Table 2 and Table 3 was 3502 (mean citations 175.1, SD: 53.7 per trial). It should be noted that three clinical trials were based on the same sample population [26,27,28]; therefore, the first published one was considered since the inclusion/exclusion criteria are the same [27].

Authors from non-clinical institutions were part of all trials except one [92], whereas authors from clinical institutions were present in 45.1% of the studies (*n* = 32/73). The total number of participants in the clinical trials were 6688 (279 men and 6409 women). Sixty-one percent of the trials (*n* = 45/73) just recruited women, and the remaining studies presented a higher number of women than men in their sample. One study failed to specify the sex [51], and another did not detail either sex or mean age [78]. Another trial did not specify the mean age [36], which ranged from 38 to 59 years for the trial.

### 3.3. Methodological Quality

According to the PEDro scale, the methodological quality scores ranged from 2 to 8 points (mean: 5.9, SD: 0.1) out of a maximum of 10 points. From the 20 articles included by citation criteria (mean: 5.6, SD: 0.1), all were rated ≥5 points, except one with a score of 4 [35]. In addition, 53 out of 61 of those published in Q1 JCR journal achieved ≥5 points (mean score: 6, SD: 0.14), while the remaining 8 trials received <5 points. There were four clinical trials without evaluation in the PEDro database. According to researchers evaluation, three obtained a good methodological quality [48,56,63].

### 3.4. Inclusion Criteria in Clinical Trials

After examining the inclusion criteria of all trials, none considered comorbidity from a visceral origin. Only two trials included the absence of concomitant somatic disorders but without any other specification [48,84]. Generally, the common criterion for most clinical trials was “Patients diagnosed with fibromyalgia according to ACR criteria”. Three clinical trials presented diagnostic criteria that differed from the ACR, such as the criteria proposed by Yunus [91], Smythe [95], or both authors [40]. In addition, five studies did not specify which criteria they used for diagnosis of FM [34,51,52,56,62].

### 3.5. Exclusion Criteria in Clinical Trials

The exclusion criteria were heterogeneous and included pregnancy [27,46,47,53,59,60,61,63,71,74,82,83,86,88,98], neurological diseases [29,35,37,39,41,44,62,65,77,81,82,84,97], rheumatic diseases [29,32,33,39,41,42,43,44,47,55,57,58,59,60,65,74,77,79,80,83,88,91,94,98], psychological/psychiatric conditions [27,32,38,42,45,46,49,50,55,59,60,61,63,64,66,69,70,73,74,75,79,80,81,82,83,89,91,94], intake medication [32,35,41,44,49,54,57,58,64,66,75,95], diabetes mellitus [32,43,44,55,57,58,65,85], cancer [32,34,46,58,64,67,82,89], skin disorders [29,50,51,52,92], trauma [59,60,61,65,83,92], hypertension [32,42,43,44,57,58,65,66,72], migraine [59,60,61,83], osteoarthritis [30,43,66,85,91], peripheral nerve entrapment [59,60,61,83], obesity [29,39,74], substance abuse [64,73,77], and hypotension [50,51,65]. It is important to mention that in 54.7% (*n* = 40/73) of clinical trials, people with cardiac, pulmonary or kidney diseases were excluded since these “visceral” conditions could limit the therapeutic intervention used in these trials (exercise). Further, seven clinical trials excluded subjects with some visceral pathologies, but not IBS [41,51,64,77,81,82,89]. Four clinical trials did not define any exclusion criteria [40,78,87,93].

## 4. Discussion

### 4.1. Findings

The current scoping review has observed that the presence of IBS, a common medical comorbidity, is not consistently considered for the selection of participants in highly cited or published in high-impact journal clinical trials investigating the effects of physical therapy in FM, which could lead to a selection bias. 

All clinical trials included individuals with a diagnosis of FM according to ACR criteria, except Buckelew et al. [91], McCain et al. [95] and Wigers et al. [40] that used other criteria. In addition, five studies [34,51,52,56,62] failed to specify the criteria by which the diagnosis of FM was made. The exclusion criteria were generally more heterogeneous, including psychological/psychiatric diseases, neurological diseases, rheumatic diseases, medication, or diabetes mellitus, among the most common. In general, the presence of visceral pathology was not summarized as an exclusion criterion. Four studies considered the possible comorbid conditions that may worsen FM symptomatology without specifying any particular visceral pathology [50,64,89,90]. In addition to IBS, it is important to highlight that other pathologies of visceral origin are also highly comorbid in FMS, and, again, they were not considered in the included trials. This should prompt us to consider the current diagnosis of FM, since it is mainly based on the presence of pain symptoms, considering these comorbidities into this complex spectrum could help to improve the quality of life and management of these patients. Interestingly, albeit the high comorbidity between IBS and FM [12,99], no clinical trial included in this scoping review commented anything on this relationship.

### 4.2. Why IBS Can Be Relevant for FMS Clinical Outcomes?

Current hypotheses support that IBS ad FMS share common underlying mechanisms leading to increased excitability of central nociceptive pathways [15]. In fact, the presence of previous IBS has been found to be the strongest predictor for new-onset FM development [100]. The presence of comorbid visceral conditions, e.g., IBS, in a musculoskeletal pain condition such as FM, should be considered in clinical practice since visceral pain enhances sensitization [101]. An exacerbation of the symptoms when two comorbid conditions exist is labeled as functional somatic syndrome [102], a situation which should be carefully explored and considered in the management of chronic pain conditions exhibiting manifestations at different levels such as those occurring in individuals with FM. In such a scenario, early recognition of comorbid syndromes of different etiology, but exhibiting a common mechanism, may identify subgroups of patients with different etiologies and different needs of treatment [103].

Comorbid visceral conditions should not be ignored when a physical therapy intervention is tested, as potentially occurred in the identified trials in the current scoping review. We do not know if considering comorbid visceral conditions in people with musculoskeletal pain conditions participating in physical therapy clinical trials could lead to potentially different clinical outcomes. This is relevant, considering that visceral pain shares several features with musculoskeletal pain but clearly requires different therapeutic strategies. For instance, the current understanding of the neurosciences is continuously evolving for better adaptation of exercise programs in individuals with nociplastic pain, a category where FM could be included [104]. It is possible that individuals with FM and comorbid IBS need different exercise programs to those with other comorbidities or without IBS. Future clinical trials should investigate the effects of multimodal therapeutic approaches considering the presence of these visceral comorbidities, e.g., IBS, in patients with a primary musculoskeletal complaint, e.g., FM.

### 4.3. Strengths and Limitations

Findings from the current scoping review should be considered according to its strengths and limitations. Strengths include a comprehensive literature search, methodological data extraction, and the inclusion of highly cited and published clinical trials in high-impact journals investigating physical therapy for FM. Among the limitations, first, the search was conducted on a single database, the WOS, because it is the only database presenting the index classification by JCR. Second, physical therapy interventions were heterogeneous, ranging from manual therapy to exercise alone or combined with physical agents. Third, 95% of the patients included in the clinical trials were female; nonetheless, this is related to the fact that FM is more prevalent in females and also that a greater frequency of comorbidity in pain syndromes is present in females [105].

## 5. Conclusions

This scoping review found that highly cited clinical trials or those published in high impact journals investigating the effects of physical therapy interventions in individuals with FM did not consider the presence of comorbid IBS in their eligibility criteria. In turn, other pathologies were sometimes considered, mostly linked to exercise, e.g., cardiac or kidney diseases. Current results highlight that the presence of intestinal pathology is underestimated when treating a musculoskeletal pain condition. Based on our results, stricter inclusion and exclusion criteria would be required in clinical trials involving patients with FM to avoid possible subject selection biases.

## Figures and Tables

**Table 1:** Clinical trials evaluating the effects of physical therapy in FM fulfilling the most-cited criterion only (*n* = 12).

Study Identification Authors’ InstitutionsNumber of Citations	PEDro Score	Objective	Participants (Number, Sex, Mean Age)	Inclusion Criteria Related with FM	Exclusion Criteria Related with Visceral Diseases
Assis et al., 2006 [29] Clinical: 0 Non-clinical: 8 Citation: 120	8/10	To compare the clinical effectiveness of aerobic exercise in the water with walking/jogging for women with FM.	*n* = 60 Sex: All women Mean Age: 43 years	Diagnostic criteria for FM by the American College of Rheumatology 1990.	Symptomatic cardiac failure; uncontrolled thyroid disturbances; coronary disease; pulmonary disease.
Burckhardt et al., 1994 [30] Clinical: 1 Non-clinical: 3 Citation: 267	5/10	To determinate the effectiveness of self-management education and physical training in decreasing FM symptoms and increasing physical and psychological well-being.	*n* = 99 Sex: All women Mean Age: 47 years	Diagnostic criteria for FM by the American College of Rheumatology 1990.	No criteria related to visceral disease.
Gowans et al., 1999 [31] Clinical: 3 Non-clinical: 1 Citation: 170	5/10	To evaluate the efficacy of a 6-week exercise and educational program for patients with FM.	*n* = 41 Sex: Women (32) Men (9) Mean Age: 45 years	Diagnostic criteria for FM by the American College of Rheumatology 1990.	No criteria related to visceral disease.
Gowans et al., 2001 [32] Clinical: 0 Non-clinical: 6 Citation: 157	7/10	To evaluate the effect of exercise on mood and physical function in individuals with FM.	*n* = 81 Sex: Women (72) Men (9) Mean age: 48 years	Diagnostic criteria for FM by the American College of Rheumatology 1990.	Diagnosed with high blood pressure or symptomatic cardiac disease; had other serious systemic diseases (e.g., systemic lupus erythematosus, cancer, diabetes).
King et al., 2002 [33] Clinical: 0 Non-clinical: 5 Citation: 125	6/10	To examine the effectiveness of a supervised aerobic exercise program, a self-management education program, and the combination of exercise and education for women with FM.	*n* = 152 Sex: All women Mean age: 46 years	Diagnostic criteria for FM by the American College of Rheumatology 1990.	Conditions that precluded ability to exercise (severe cardiac arrhythmia, severe shortness of breath).
Lorig et al., 2008 [34] Clinical: 0 Non-clinical: 4 Citation: 241	5/10	To determine the efficacy of an internet-based arthritis self-management program (ASMP) as a resource for arthritis patients unable or unwilling to attend small-group ASMPs, which have proven effective in changing health-related behaviors and improving health status measures.	*n* = 855 Sex: Women (781) Men (74)Mean age: 52 years	Diagnosis of osteoarthritis, rheumatoid arthritis, or FM.	No criteria related to visceral disease.
Martin et al., 1996 [35] Clinical: 0 Non-clinical: 6 Citation: 179	4/10	To assess the utility of an exercise program, which included aerobic, flexibility and strengthening elements, in the treatment of FM. FM is a chronic musculoskeletal condition characterized by diffuse musculoskeletal pain and aching. It has been suggested that aerobic exercise is helpful in its treatment.	*n* = 60 Sex: Women (58) Men (2) Mean age: 45 years	Diagnostic criteria for FM by the American College of Rheumatology 1990.	Cardiovascular, pulmonary, or renal disease that precluded involvement in an exercise program.
Redondo et al., 2004 [36] Clinical: 7 Non-clinical: 2 Citation: 142	6/10	To analyze the long-term efficacy of 2 interventions for female FM patients: (1) cognitive behavioral therapy (CBT), and (2) a physical exercise (PE)–based strategy.	*n* = 56 Sex: All women Mean age: Not described	Diagnostic criteria for FM by the American College of Rheumatology 1990.	Serious concomitant disease.
Richards et al., 2002 [37] Clinical: 1 Non-clinical: 1 Citation: 153	5/10	To evaluate cardiovascular fitness exercise in people with FM.	*n* = 136 Sex: Women (126) Men (10) Mean age: 47 years	Diagnostic criteria for FM by the American College of Rheumatology 1990.	Alternative medical diagnosis could explain current symptoms; severe pulmonary, cardiovascular or renal disease precluding involvement in aerobic exercise or inability to cooperate.
Sephton et al., 2007 [38] Clinical: 0 Non-clinical: 7 Citation: 182	5/10	To test the effects of the mindfulness-based stress reduction (MBSR) intervention on depressive symptoms in patients with FM.	*n* = 91 Sex: All women Mean age: 48 years	Diagnostic criteria for FM by the American College of Rheumatology 1990.	No criteria related to visceral disease.
Valim et al., 2003 [39] Clinical: 1 Non-clinical: 7 Citation: 146	5/10	To compare 2 exercise modalities, aerobic fitness training and stretching exercises, in patients with FM in relation to function, pain, quality of life, depression, and anxiety, and to correlate the cardiorespiratory fitness gain with symptom improvement.	*n* = 76 Sex: All women Mean age: 47 years	Diagnostic criteria for FM by the American College of Rheumatology 1990.	Cardiorespiratory diseases limiting their physical activities, hypothyroidism.
Wigers et al., 1996 [40] Clinical: 1 Non-clinical: 2 Citation: 227	6/10	To determine and compare short- and long-term effects of aerobic exercise, stress management treatment, and treatment as usual in FM	*n* = 60 Sex: Women (55) Men (5) Mean age: 44 years	To meet and fulfil the diagnostic criteria of both Smythe and Yunus et al.	No exclusion criteria were specified

**Table 2:** Clinical trials evaluating the effects of physical therapy in FM fulfilled the Q1-JCR criterion only (*n* = 53).

Study Identification Authors’ Institutions Number of Citations	PEDro Score	Objective	Participants (Number, Sex, Mean Age)	Inclusion Criteria Related with FM	Exclusion Criteria Related with Visceral Diseases
Almeida et al., 2003 [41] Clinical: 0 Non-clinical: 4 Citation: 57	6/10	To assess the effects of combined therapy with pulsed ultrasound and interferential current on pain and sleep in FM.	*n* = 17 Sex: All women Mean age: 57 years	Diagnostic criteria for FM by the American College of Rheumatology 1990.	Showed evidence of endocrine disease.
Altan et al., 2009 [42] Clinical: 0 Non-clinical: 4 Citation: 77	6/10	To investigate the effects of Pilates on pain, functional status, and quality of life in FM, which is known to be a chronic musculoskeletal disorder.	*n* = 50 Sex: All women Mean age: 49 years	Diagnostic criteria for FM by the American College of Rheumatology 1990.	Severe cardiopulmonary problems.
Andrade et al., 2017 [43] Clinical: 0 Non-clinical: 6 Citation: 4	6/10	To investigate whether APT can help improve body composition and increase the aerobic functional capacity in women with FMS, and whether oxygen uptake (VO^2^) related to LBM can better quantify the functional capacity of this population.	*n* = 54 Sex: All women Mean age: 48 years	Diagnostic criteria for FM by the American College of Rheumatology 1990 and 2010.	Systemic uncontrolled diseases (e.g., hypertension); cardiovascular system abnormalities.
Andrade et al., 2019 [44] Clinical: 0 Non-clinical: 5 Citation: 7	8/10	To evaluate variable oxygen uptake (VO2) relative to lean body mass and clinical symptomatology in women with FMS submitted to APT and after 16 weeks of detraining period, and to evaluate the association between the magnitude of VO^2^ improvement relative to LBM and clinical symptomatology.	*n* = 54 Sex: All women Mean age: 48 years	Diagnostic criteria for FM by the American College of Rheumatology 1990 and 2010.	Cardiovascular diseases; systemic arterial hypertension; arrhythmias.
Bjersing et al., 2012 [45] Clinical: 4 Non-clinical: 1 Citation: 21	4/10	To examine changes in serum IGF-1, cerebrospinal fluid (CSF), neuropeptides, and cytokines during aerobic exercise in FM patients.	*n* = 49 Sex: All women Mean age: 52 years	Diagnostic criteria for FM by the American College of Rheumatology 1990.	No criteria related to visceral disease.
Bourgault et al., 2015 [46] Clinical: 0 Non-clinical: 8 Citation: 23	5/10	To evaluate the efficacy of the PASSAGE Program, a structured multicomponent interdisciplinary group intervention for the self-management of FMS.	*n* = 56 Sex: Women (52) Men (4) Mean age: 48 years	Diagnostic criteria for FM by the American College of Rheumatology 1990.	Uncontrolled metabolic disease.
Carbonario et al., 2013 [47] Clinical: 0 Non-clinical: 4 Citation: 21	4/10	To assess the efficacy of high-frequency transcutaneous electrical nerve stimulation (TENS) as an adjuvant therapy to aerobic and stretching exercises, for the treatment of FM.	*n* = 28 Sex: All women Mean age: 52 years	Diagnostic criteria for FM by the American College of Rheumatology 1990.	Use of pacemaker; heart disease (cardiopathy or disorders of the heart rhythm).
Carbonell-Baeza et al., 2011 [48] Clinical: 0 Non-clinical: 7 Citation: 47	5/10	To determine the effects of a 3-month multidisciplinary intervention on pain (primary outcome), body composition and physical fitness (secondary outcomes) in women with FM.	*n* = 75 Sex: All women Mean age: 51 years	Diagnostic criteria for FM by the American College of Rheumatology 1990.Not to have any other severe somatic disorders; diseases that prevent physical loading.	No criteria related to visceral disease.
Carson et al., 2010 [49] Clinical: 0 Non-clinical: 6 Citation: 116	7/10	To evaluate the effects of a comprehensive yoga intervention on FM symptoms and coping.	*n* = 53 Sex: All women Mean age: 54 years	Diagnostic criteria for FM by the American College of Rheumatology 1990.	No criteria related to visceral disease.
Castro-Sánchez et al., 2018 [50] Clinical: 1 Non-clinical: 6 Citation: 4	8/10	To compare the effectiveness of dry needling versus myofascial release on myofascial trigger point pain in cervical muscles, quality of life, impact of symptom pain, quality of sleep, anxiety, depression, and fatigue in patients with FM syndrome.	*n* = 64 Sex: Women (58) Men (6) Mean age: 47 years	Diagnostic criteria for FM by the American College of Rheumatology 2010.	Presence of cardiac, renal or hepatic insufficiency; comorbid condition; hypotension.
Castro-Sánchez et al., 2011 [51] Clinical: 1 Non-clinical: 5 Citation: 42	6/10	To determine the effect of myofascial release techniques on pain symptoms, postural stability and physical function in FM syndrome.	*n* = 86 Sex: Not described Mean age: 54 years	Not defined.	Hypotension; treatment-limiting respiratory disorders.
Castro-Sánchez et al., 2011 [52] Clinical: 1 Non-clinical: 5 Citation: 34	8/10	To determine the effects of craniosacral therapy on sensitive tender points and heart rate variability in patients with FM.	*n* = 92 Sex: All women Mean age: 52 years	Not defined.	No criteria related to visceral disease.
Collado-Mateo et al., 2017 [53] Clinical: 0 Non-clinical: 5 Citation: 16	7/10	To evaluate the effects of an exergame-based intervention in a population sample of women with FM.	*n* = 83 Sex: All women Mean age: 52 years	Diagnostic criteria for FM by the American College of Rheumatology 1990 and 2010.	Contraindications for physical exercise.
Da costa et al., 2005 [54] Clinical: 0 Non-clinical: 7 Citation: 78	8/10	To determine the efficacy of a 12-week individualized home-based exercise program on physical functioning, pain severity and psychological distress for women with FM.	*n* = 79 Sex: All women Mean age: 51 years	Diagnostic criteria for FM by the American College of Rheumatology 1990.	Concomitant diseases which precluded participation in exercise.
Fernandes et al., 2016 [55] Clinical: 0 Non-clinical: 5 Citation: 12	8/10	To evaluate the effect of swimming on pain, functional capacity, aerobic capacity and quality of life on patients with FM.	*n* = 75 Sex: All women Mean age: 49 years	Diagnostic criteria for FM by the American College of Rheumatology 1990.	Uncontrolled cardiorespiratory disease, any health condition for which physical exercise was contraindicated.
Garrido-Ardila et al., 2020 [56] Clinical: 0 Non-clinical: 5 Citation: 3	7/10	To investigate the effectiveness of a core stability training physiotherapy program vs. acupuncture for the management of balance and functional capacity impairments of women with FM.	*n* = 135 Sex: All women Mean age: 55 years	Not defined.	No criteria related to visceral disease.
Gavi et al., 2014 [57] Clinical: 2 Non-clinical: 5 Citations: 36	4/10	To assess the chronic effects of strengthening exercises (STRE) on autonomic modulation, pain perception and the quality of life of FM patients.	*n* = 80 Sex: All women Mean age: 46 years	Diagnostic criteria for FM by the American College of Rheumatology 1990.	Cardiovascular, respiratory, and metabolic diseases that could limit exercise; diseases associated with autonomic dysfunction, such as arterial hypertension and coronary insufficiency; positive treadmill test for myocardial ischemia.
Gowans et al., 2002 [58] Clinical: 0 Non-clinical: 3 Citation: 28	5/10	To compare scales measuring exercise-induced changes in mood.	*n* = 50 Sex: Women (44) Men (6) Mean age: 47 years	Diagnostic criteria for FM by the American College of Rheumatology 1990.	High blood pressure or symptomatic cardiac disease; other serious systemic diseases (e.g., systemic lupus erythematosus, cancer, diabetes).
Gusi and Tomas-Carus, 2008 [59] Clinical: 0 Non-clinical: 2 Citation: 4	5/10	To assess the cost-utility of adding an aquatic exercise program to the usual care of women with FM.	*n* = 33 Sex: All women Mean age: 51 years	Diagnostic criteria for FM by the American College of Rheumatology 1990.	Diseases that prevent physical loading.
Gusi et al., 2006 [60] Clinical: 1 Non-clinical: 4 Citation: 108	5/10	To evaluate the short- and long-term efficacy of exercise therapy in a warm, waist-high pool in women with FM.	*n* = 35 Sex: All women Mean age: 51 years	Diagnostic criteria for FM by the American College of Rheumatology 1990.	No criteria related to visceral disease.
Gusi et al., 2010 [61] Clinical: 1 Non-clinical:4 Citation: 28	7/10	To evaluate the feasibility and efficacy of tilt whole-body vibration (WBV) for improving dynamic balance in women with FM.	*n* = 41 Sex: All women Mean age: 53 years	Diagnostic criteria for FM by the American College of Rheumatology 1990.	Diseases that prevent physical loading.
Hoeger Bement et al., 2011 [62] Clinical: 0 Non-clinical: 6 Citation: 41	4/10	To identify exercise protocols incorporating isometric contractions that provide pain relief in women with FM.	*n* = 15 Sex: All women Mean age: 52 years	Not defined.	Screened for known cardiopulmonary and neurologic problems
Hooten et al., 2012 [63] Clinical: 0 Non-clinical: 4 Citation: 71	7/10	The primary aim of this randomized equivalence trial involving patients with FM admitted to an interdisciplinary pain treatment program was to test the hypothesis that strengthening (*n* = 36) and aerobic (*n* = 36) exercise have equivalent effects (95% confidence interval within an equivalence margin ±8) on pain, as measured by the pain severity subscale of the Multidimensional Pain Inventory.	*n* = 72 Sex: Women (65) Men (7) Mean age: 46 years.	Diagnostic criteria for FM by the American College of Rheumatology 1990.	Cardiovascular, pulmonary or other systemic disease that could limit strength training or aerobic conditioning.
Hsu et al., 2010 [64] Clinical: 1 Non-clinical: 5 Citation: 48	7/10	To evaluate an innovative, affective self-awareness (ASA) intervention, which was designed to reduce pain and improve functioning in individuals with FM.	*n* = 45 Sex: All women Mean age: 50 years	Diagnostic criteria for FM by the American College of Rheumatology 1990.	Serious comorbid medical conditions that could confound the influence of FM in the next 6 months (e.g., cancer, heart disease).
Kibar et al., 2015 [65] Clinical: 0 Non-clinical: 5 Citation: 17	5/10	To determine the effectiveness of balance exercises on the functional level and quality of life of patients with FM syndrome and investigate the circumstances associated with balance disorders in FMS.	*n* = 57 Sex: Women (54) Men (3) Mean age: 48 years	Diagnostic criteria for FM by the American College of Rheumatology 2010.	Advanced cardiovascular or lung pathologies and those with uncontrolled hypertension or hypotension.
Larsson et al., 2015 [66] Clinical: 2 Non-clinical: 7 Citation: 51	7/10	To examine the effects of a progressive resistance exercise program on muscle strength, health status, and current pain intensity in women with FM.	*n* = 130 All women 51 years	Diagnostic criteria for FM by the American College of Rheumatology 1990.	Comorbidity defined by anamnesis; high blood pressure (>160/90 mmHg); other severe somatic disorders.
Lemstra and Olszynski, 2005 [67] Clinical: 0 Non-clinical: 2 Citation: 114	8/10	To assess the effectiveness of multidisciplinary rehabilitation in the treatment of FM in comparison to standard medical care.	*n* = 79 Sex: Women (67) Men (12) Mean age: 49 years	Diagnostic criteria for FM by the American College of Rheumatology 1990.	No criteria related to visceral disease.
Lynch et al., 2012 [68] Clinical: 0 Non-clinical: 4 Citation: 41	6/10	To compare the effects of self-practice of qigong (45 min daily, eight weeks) with a control group over a six-month interval.	*n* = 100 Sex: Women (96) Men (4) Mean age: 52 years	Diagnostic criteria for FM by the American College of Rheumatology 1990.	Significant medical disorder that the study physician thought would compromise participant safety.
Martín et al., 2014 [69] Clinical: 2 Non-clinical: 5 Citation: 16	4/10	This study assessed the efficacy of a 6-week interdisciplinary treatment that combines coordinated PSYchological, Medical, Educational, and PHYsiotherapeutic interventions (PSYMEPHY) compared with standard pharmacologic care.	*n* = 153 Sex: Women (143) Men (10) Mean age: 50 years	Diagnostic criteria for FM by the American College of Rheumatology 1990.	Suffering from a severe organic disorder.
Martín et al., 2014 [70] Clinical: 6 Non-clinical: 1 Citation: 6	4/10	To assess the effects of an interdisciplinary treatment for FM on patients’ physical and psychosocial parameters.	*n* = 110 Sex: Women (100) Men (10) Mean age: 50 years	Diagnostic criteria for FM by the American College of Rheumatology 1990.	Suffering from a severe organic disorder
Martín-Martínez et al., 2019 [71] Clinical: 0 Non-clinical: 5 Citation: 9	7/10	To evaluate the effects of 24-week exergame intervention in the physical fitness of women with FM in both single- and dual-task conditions.	*n* = 55 Sex: All women Mean age: 54 years	Diagnostic criteria for FM by the American College of Rheumatology 2010.	No criteria related to visceral disease.
Meyer and Lemley, 2000 [72] Clinical: 0 Non-clinical: 2Citation: 82	2/10	To examine the effect of a 24-wk walking program at predetermined intensities on FM.	*n* = 21 Sex: All women Mean age: 50 years	Diagnostic criteria for FM by the American College of Rheumatology 1990.	Uncontrolled hypertension; history of heart or respiratory disease.
Molinari et al., 2018 [73] Clinical: 1 Non-clinical: 5 Citation: 5	4/10	To test the efficacy of the best possible self intervention using information and communication technologies with FM patients.	*n* = 71 Sex: All women Mean age: 51 years	Diagnostic criteria for FM by the American College of Rheumatology 1990 and 2010.	No criteria related to visceral disease.
Munguía et al., 2008 [74] Clinical: 0 Non-clinical: 2 Citation: 74	7/10	To evaluate the effects of a 16-week exercise therapy in a chest-high pool of warm water through applicable tests in the clinical practice on the global symptomatology of women with FM and to determine exercise adherence levels.	*n* = 60 Sex: All women Mean age: 48 years	Diagnostic criteria for FM by the American College of Rheumatology 1990.	Known cardiopulmonary diseases; endocrine disturbances uncontrolled.
Newcomb et al., 2011 [75] Clinical: 1 Non-clinical: 3 Citation: 39	5/10	The purpose of this study was to examine the influence of a preferred- versus a prescribed-intensity exercise session on pain in women with FM.	*n* = 21 Sex: All women Mean age: 44 years	Diagnostic criteria for FM by the American College of Rheumatology 1990.	No criteria related to visceral disease.
Oliver et al., 2001 [76] Clinical: 0 Non-clinical: 4 Citation: 58	5/10	We carried out social support and education interventions with patients with FM and assessed the effect on health care costs, psychosocial variables, and health status.	*n* = 600 Sex: Women (572) Men (28) Mean age: 54 years	Diagnostic criteria for FM by the American College of Rheumatology 1990.	No criteria related to visceral disease.
Pujol et al., 2019 [77] Clinical: 8 Non-clinical: 1 Citation: 0	7/10	To test the effect of vibrotactile stimulation on symptom relief in FM patients.	*n* = 63 Sex: All women Mean age: 54 years	Diagnostic criteria for FM by the American College of Rheumatology 1990 and 2010. Did not suffer from any other disorder that might account for the pain.	Severe or non-stable medical or endocrine, disorder.
Ramsay et al., 2000 [78] Clinical: 4 Non-clinical: 2 Citation: 77	6/10	To compare a supervised 12-week aerobic exercise class with unsupervised home aerobic exercises in the treatment of patients with FM.	*n* = 74 Sex: - Mean age: -	Diagnostic criteria for FM by the American College of Rheumatology 1990.	No exclusion criteria were specified
Sañudo et al. 2010 [79] Clinical: 0 Non-clinical: 6 Citation: 59	6/10	To investigate the effects of supervised aerobic exercise and a combined program of supervised aerobic, muscle strengthening, and flexibility exercises (combined exercise) on important health outcomes in women with FM syndrome.	*n* = 64 Sex: All women Mean age: 56 years	Diagnostic criteria for FM by the American College of Rheumatology 1990.	Presence of respiratory or cardiovascular diseases.
Sañudo et al., 2011 [80] Clinical: 0 Non-clinical: 5 Citation: 41	8/10	To assess the impact of a long-term exercise program vs. usual care on perceived health status, functional capacity and depression in patients with FM.	*n* = 42 Sex: All women Mean age: 56 years	Diagnostic criteria for FM by the American College of Rheumatology 1990.	Any significant concomitant medical illness, such as respiratory or cardiovascular diseases that would prevent physical exercise.
Targino et al., 2008 [81] Clinical: 0 Non-clinical: 8 Citation: 39	7/10	To evaluate the effectiveness of acupuncture for FM.	*n* = 58 Sex: All women Mean age: 51 years	Diagnostic criteria for FM by the American College of Rheumatology 1990.	Cardiac disease.
Thieme et al., 2003 [82] Clinical: 0 Non-clinical: 3 Citation: 114	5/10	To evaluate the efficacy of operant pain treatment for FM syndrome in an inpatient setting.	*n* = 61 Sex: All women Mean age: 47 years	Diagnostic criteria for FM by the American College of Rheumatology 1990.	Severe disease such as a liver, or renal disease.
Tomas-Carus et al., 2007 [83] Clinical: 2 Non-clinical: 4 Citation: 84	5/10	To evaluate the effects of a 12-week period of aquatic training and subsequent detraining on health-related quality of life and physical fitness in females with FM.	*n* = 34 Sex: All women Mean age: 51 years	Diagnostic criteria for FM by the American College of Rheumatology 1990.	Diseases that might prevent physical loading.
Torres et al., 2015 [84] Clinical: 0 Non-clinical: 6 Citation: 5	7/10	To examine the effects of an active neurodynamic mobilization program on pain, neurodynamics, perceived health state, and fatigue in patients with FM syndrome.	*n* = 48 Sex: Women (39) Men (9) Mean age: 53 years	Diagnostic criteria for FM by the American College of Rheumatology 2010.Did not suffer from concomitant somatic disorders.	No criteria related to visceral disease.
Valkeinen et al., 2008 [85] Clinical: 2 Non-clinical: 4 Citation: 55	6/10	To examine the effectiveness of concurrent strength and endurance training on muscle strength, aerobic and functional performance, and symptoms in postmenopausal women with FM.	*n* = 26 Sex: All women Mean age: 59 years	Diagnostic criteria for FM by the American College of Rheumatology 1990.	Severe cardiovascular disease; disorders of thyroid gland; any other diseases that might confound the results of the study.
Van Koulil et al., 2010 [27] Clinical: 1 Non-clinical: 12 Citation: 90	6/10	To select patients at risk of long-term dysfunction and offering tailored treatment may be promising for beneficial treatment effects	*n* = 158 Sex: Women (148) Men (10) Mean age: 41 years	Diagnostic criteria for FM by the American College of Rheumatology 1990.	No criteria related to visceral disease.
Van Koulil et al., 2011 [26] Clinical: 0 Non-clinical: 11 Citation: 17	6/10	To examine the cognitive–behavioral mechanisms of a pain-avoidance treatment and a pain-persistence treatment.	*n* = 158 Sex: Women (148) Men (10) Mean age: 41 years	Diagnostic criteria for FM by the American College of Rheumatology 1990.	No criteria related to visceral disease.
Van Koulil et al., 2011 [28] Clinical: 0 Non-clinical: 11Citation: 19	5/10	To propose that a tailored treatment approach might yield more promising treatment outcomes.	*n* = 158 Sex: Women (148) Men (10) Mean age: 41 years	Diagnostic criteria for FM by the American College of Rheumatology 1990.	No criteria related to visceral disease.
Villafaina et al., 2019 [86] Clinical: 0 Non-clinical: 5 Citation: 6	7/10	To evaluate the effects of a 24-week exergame-based intervention on health-related quality of life and pain in patients with FM as well as to analyze the effectiveness of the intervention in subgroups of patients with different pain intensity levels.	*n* = 55 Sex: All women Mean age: 54 years	Diagnostic criteria for FM by the American College of Rheumatology 2010.	Contraindications for physical exercise programs.
Vitorino et al., 2006 [87] Clinical: 0 Non-clinical: 3 Citation: 44	8/10	To compare hydrotherapy (HT) and conventional physiotherapy (CP) in the treatment of FM, regarding quality of life, total sleep time (TST), and total nap time (TNT).	*n* = 50 Sex: All women Mean age: 48 years	Diagnostic criteria for FM by the American College of Rheumatology 1990.	No exclusion criteria were specified
Wang et al., 2018 [88] Clinical: 1 Non-clinical: 9 Citation: 37	7/10	To determine the effectiveness of tai chi interventions compared with aerobic exercise, a current core standard treatment in patients with FM, and to test whether the effectiveness of tai chi depends on its dosage or duration.	*n* = 226 Sex: Women (209) Men (17) Mean age: 52 years	Diagnostic criteria for FM by the American College of Rheumatology 1990 and 2010.Not have a disorder that would otherwise explain the pain.	Serious medical conditions that might limit their participation.
Williams et al., 2010 [89] Clinical: 2 Non-clinical: 4 Citation: 105	7/10	To evaluate the incremental utility of adding an internet-based behavioral self-management program to the standard care of individuals with FM.	*n* = 118 Sex: Women (112) Men (6) Mean age: 50 years	Diagnostic criteria for FM by the American College of Rheumatology 1990.	Comorbid medical illnesses capable of causing a worsening of physical functional status independent of FM (e.g., cardiopulmonary disorders, uncontrolled endocrine or allergic disorders.
Zijlstra et al., 2005 [90] Clinical: 5 Non-clinical: 1 Citation: 91	5/10	To study the effect of a combination of thalassotherapy, exercise and patient education in people with FM.	*n* = 134 Sex: Women (128) Men (6) Mean age: 48 years	Diagnostic criteria for FM by the American College of Rheumatology 1990.	Comorbidity interfering with spa treatment; other serious comorbidity.

**Table 3:** Clinical trials evaluating the effects of physical therapy in FM fulfilling both criteria (*n* = 8).

Study Identification Authors’ Institutions Number of Citations	PEDro Score	Objective	Participants (Number, Sex, Mean Age)	Inclusion Criteria Related with FM	Exclusion Criteria Related with Visceral Diseases
Buckelew et al., 1998 [91] Clinical: 0 Non-clinical: 13 Citation: 169	6/10	To compare the effectiveness of biofeedback/relaxation, exercise, and a combined program for the treatment of FM.	*n* = 119 Sex: Women (108) Men (11) Mean age: 44 years	Yunus’ criteria require: (1) a minimum of 5 of 20 possible tender points, (2) generalized aches and pains or prominent stiffness involving at least 3 anatomic sites, and (3) the presence of at least 3 out of 10 possible minor criteria. Yunus’ minor criteria include the following: (1) modulation of symptoms by physical activity, (2) modulation of symptoms by weather factors, (3) modulation of symptoms by anxiety or stress, (4) poor sleep, (5) general fatigue, (6) anxiety, (7) chronic headaches, (8) irritable bowel syndrome, (9) subjective swelling, and (10) numbness.	Unstable or uncontrolled medical condition.
Cedraschi et al., 2004 [92] Clinical: 8 Non-clinical: 0 Citation: 137	5/10	To evaluate the efficacy of a treatment program for patients with FM based on self-management, using pool exercises and education.	*n* = 164 Sex: Women (152) Men (12) Mean age: 49 years	Diagnostic criteria for FM by the American College of Rheumatology 1990.	Prevented physical activity (for example, cardiovascular problems).
Häkkinen et al., 2001 [93] Clinical: 2 Non-clinical: 2 Citation: 120	5/10	To investigate the effects of 21 weeks’ progressive strength training on neuromuscular function and subjectively perceived symptoms in premenopausal women with FM.	*n* = 33 Sex: All women Mean age: 38 years	Diagnostic criteria for FM by the American College of Rheumatology 1990.	Not described.
Mannerkorpi et al. 2000 [94] Clinical: 1 Non-clinical: 3 Citation: 188	5/10	To evaluate the effects of 6 months of pool exercise combined with a 6-session education program for patients with FM syndrome.	*n* = 58 Sex: All women Mean Age: 46 years	Diagnostic criteria for FM by the American College of Rheumatology 1990.	Severe somatic disease.
McCain et al., 1988 [95] Clinical: 0 Non-clinical: 4 Citation: 308	5/10	To assess the effects of supervised strenuous exercise on the clinical manifestations of the fibrositis/FM syndrome.	*n* = 42 Sex: All women Mean age: 42.2 years	Smythe criteria, that is: (1) widespread aching of more than 3 months duration in more than 3 anatomic sites, (2) local tenderness at 12 of 14 specified fibrositic tender points, (3) disturbed sleep with morning fatigue and stiffness, (4) absence of traumatic, neurologic, muscular, infectious, osseous. endocrine, or other rheumatic conditions, and (5) normal Wintrobe erythrocyte sedimentation rate, creatinine phosphokinase level, latex fixation test results, antinuclear antibody factor, and thyroid-stimulating hormone level.	History of ischemic heart disease; symptomatic cardiac arrhythmia; chest pain; or exercise-induced bronchospasm.
Rooks et al., 2007 [96] Clinical: 4 Non-clinical: 5 Citation: 126	7/10	To evaluate and directly compare the effects of four common self-management interventions on well-established measures of functional status, symptom severity, and self-efficacy in women with FM.	*n* = 207 Sex: All women Mean age: 50 years	Diagnostic criteria for FM by the American College of Rheumatology 1990.	Medical conditions that limited a person’s ability to perform the exercise protocol or for whom moderate-level exercise was inadvisable.
Schachter et al., 2003 [97] Clinical: 0 Non-clinical: 4 Citation: 117	5/10	(1) To assess the effectiveness of a 16-week progressive program of home-based, video-tape-based, low-impact aerobic exercise on physical function and signs and symptoms of FM in previously sedentary women aged 20 to 55 years and (2) to compare the effects of one long exercise bout versus two short exercise bouts per training day (fractionation) on physical function, signs and symptoms of FM, and exercise adherence.	*n* = 143 Sex: All women Mean age: 42 years	Diagnostic criteria for FM by the American College of Rheumatology 1990.	More than two coronary artery disease factors outlined in the 1995 guidelines of the American College of Sports Medicine (ACSM); known cardiovascular or respiratory disease; metabolic, condition that would interfere with performance of moderate-intensity aerobic exercise.
Wang et al., 2010 [98] Clinical: 1 Non-clinical: 7 Citation:228	7/10	To compare the physical and psychological benefits of tai chi with those of a control intervention that consisted of wellness education and stretching.	*n* = 66 Sex: Women (57) Men (9) Mean Age: 55 years	Diagnostic criteria for FM by the American College of Rheumatology 1990.	Serious medical conditions that might limit their participation; those with other diagnosed medical conditions known to contribute to FM symptoms, such as thyroid disease; Sjögren’s syndrome
